# Discovery of vaccine-like recombinant SARS-CoV-2 circulating in human

**DOI:** 10.1186/s12985-022-01945-5

**Published:** 2022-12-08

**Authors:** Daniel Chang He, Cheng-Qiang He

**Affiliations:** 1 Dongying Institute, Shandong Normal University, Dongying, 257000 China; 2 Shandong Provincial Key Laboratory of Animal Resistance Biology, College of Life Science, Shandong Normal University, Jinan, 250014 Shandong Province China; 3 International Department, High School Attached to Shandong Normal University, Jinan, 250014 China

## Abstract

For viral diseases, vaccination with live attenuated vaccine (LAV) is one of the most effective means for fighting the diseases. However, LAV occasionally overflows from vaccinated individuals circulate in the population with unforeseen consequences. Currently, SARS-CoV-2 LAVs are undergoing clinical trials. In this study, we found that the viruses isolated from Indian SARS CoV-2 infected persons may be candidate LAV-derived strains, indicating the risk of SARS-CoV-2 LAV spillover from vaccinated persons, increasing the complexity of SARS-CoV-2 detection. In addition, the property of frequent recombination of SARS-CoV-2 increases the chance of LAV virulence reversion. Therefore, how to distinguish the LAV viruses from the wild strain and how to avoid the recombination of the circulating vaccine strain and the wild strain are the challenges currently faced by SARS CoV-2 LAV development.

## Introduction

Since the outbreak at the end of 2019, the novel coronavirus pneumonia (COVID-19) has continued to rage around the world, seriously endangering human health and life. According to the World Health Organization (WHO), as of May 30, 2022, more than 520 million people have suffered from the disease, with over 6.28 million deaths (https://covid19.who.int/). Moreover, these numbers keep growing. Its pathogen is acute respiratory syndrome coronavirus 2 (SARS-CoV-2), which belongs to the genus *Betacoronavirus* of the family *Coronaviridae* [3, 14]. The genome of SARS-CoV-2 is positive sense single-stranded RNA up to 30,000 nucleotides, encoding a complex system of structural and non-structural proteins [30], such as spike protein (S), envelope protein (E), nucleocapsid protein (N), and RNA polymerase [1]. Of them, the S protein, exposed on the surface of the virion, is responsible for binding the receptor protein, angiotensin-converting enzyme 2 (ACE2), and mediates the entry of the virus into the host cell after being cleaved into S1 and S2 by the Furin enzyme of the host cell [23]. In addition, S protein is also the protective antigen of SARS-CoV-2, which can induce the host to produce neutralizing antibodies and terminate its replication of in the host [5].

Vaccination remains the primary means of controlling COVID-19 although different measures are being developed to prevent and treat the pandemic. So far, a large number of vaccines have been used in clinical or are being developed, including inactivated and live attenuated vaccines, recombinant protein vaccines or recombinant subunit vaccines, nucleoid vaccines, and viral vector vaccines. Li and his colleagues reviewed the research progress of these vaccines [15]. An ideal SARS-CoV-2 vaccine should: elicit strong humoral and cellular immune responses; have equipment that is easy to store and transport; and be affordable for all countries, especially low- and middle-income countries. Although various SARS-CoV-2 vaccines have been extensively studied and used [21], rapid antigenic drift and epitope loss greatly compromises their effectiveness [25]. SARS-CoV-2 live attenuated vaccines (LAV) still have their unique advantages: activate all types of host immune responses (cellular, humoral and innate); present all epitopes to the host immune cells, thereby inducing a broad host immune response and avoiding the immune escape caused by antigenic drift as much as possible; and have relatively low storage, transportation and immune costs [4, 6, 19]. These advantages make LAV especially suitable for less developed regions and countries. Therefore, vaccine research institutions in different countries, including India and the United States (US), are adopting various strategies to construct SARS-CoV-2 LAV [21]. In July 2021, a group from the US reported the efficacy and safety of a candidate SARS-CoV-2 LAV constructed through a deoptimization strategy [28]. In the spike (S) protein gene of this strain, besides the deletion of the segment encoding for the furin cleavage site, 283 point mutations were introduced. Although these artificial mutations did not change the amino acid sequence, the variant is highly attenuated. Its antigen epitope is a perfect match to that of the circulating wild-type (WT) strain, providing the capacity for a broad immune response and making the vaccine more likely to retain efficacy. In Vero E6 cells, COVI-VAC is temperature sensitive and has high replication titer. After COVI-VAC vaccination, Syrian golden hamsters did not show significant pathological changes. In vitro, the sera of immunized hamsters could neutralize the WT virus. In vivo, when hamsters were challenged with the WT virus, COVI-VAC vaccination reduced viral titers in the lung, rendered the virus undetectable in the brain, and protected hamsters from almost all virus-associated weight loss. Moreover, a single intranasal dose could provide enough protection for the inoculated animals. These advantages endow COVI-VAC with the promise of mass vaccination. Groups from many countries have also reported their progress in the research of LAV [16, 24, 26].

As a pathogen that is prone to homologous recombination between viruses [12, 13, 27], SARS-CoV-2 LAV virus spilled from the vaccinated population into the environment will inevitably recombine with circulating strains, resulting in new circulating strains with unpredictable consequences. Therefore, knowing whether LAV spill over into the environment from vaccinated individuals is an essential for the safety assessment of LAV. So far, there have been no reports in this regard. India, one of the largest developing countries, is conducting research on LAV [21]. Therefore, in this study, we analyzed the genome sequences of SARS-CoV-2 isolated from infected persons in India before July 2021 in the SARS-CoV-2 databases, to explore the possibility of spillover of LAV so as to provide references for the study of LAV.

## Materials and methods

### Virus sequences

From the SARS-CoV-2 database of GenBank or GISAID, we collected the genome sequences of 1643 SARS-CoV-2 isolated from infected individuals in India before August 2021. With the help of the MUSCLE program in the MEGA X software package [11], sequence alignments were performed on these sequences, and the optimized alignment results were finally obtained for subsequent analysis.

### Recombination analysis

In order to determine whether there is some viruses undergoing genetic recombination in their genome, the recombination analysis software RDP 3.0 [20] was used to analyze the above processed data set to preliminarily screen recombinant sequences. And then, the SimPlot program [18] was used to visualize the genomic sequence similarity between the putative recombinant and their potential parental virus so as to further determine the reliability of the recombination signal.

### Phylogenetic analysis

To determine the phylogeny of viruses with recombinant signals, we downloaded the LAV strain and other reference viruses of different genotypes from the SARS-CoV-2 database (Table 1) and analyzed their phylogenetic history. Before the phylogenetic reconstruction, the nucleotide substitution model selection tool MODELS in the phylogenetic analysis software package MEGA X [11] was used to find the optimal substitution model, and then, the maximum likelihood method was used to reconstruct phylogenetic history employing the optimal substitution model. The robustness of the most recent common ancestor of each phylogenetic branch was determined by the bootstrap method of 1000 replications, and the bootstrap value > 70% was regarded as robustness.

**Table 1:** Representative isolates analyzed in this study

Accession ID	Variant	Pango lineage	Host	Location	Collection date	Sequencing technology	Submitter
EPI_ISL_3795015	Alpha, V1	B.1.1.7	Human	Sweden	2021/05/17	Ultraplex	Öhman et al.
EPI_ISL_4648355	Beta, V2	B.1.351	Human	Hong Kong	2021/04	Illumina	Gu et al.
EPI_ISL_4508779	Gamma, V3	P.1	Human	USA	2021/07/25	Illumina	Smith et al.
EPI_ISL_5104539	Delta	B.1.617.2	Human	India	2021/09/07	Illumina	Yadav et al.
EPI_ISL_8097272	Omicron, 21 K	BA.1	Human	Botswana	2021/12/08	Nanopore	Moyo et al.
EPI_ISL_11324640	Omicron, 21 L	BA.2	Human	India	2022/01/04	Illumina	Kumar et al.
EPI_ISL_13258801	Omicron, 22 A	BA.4	Human	USA	2022/05/03	Illumina	Howard et al.
EPI_ISL_12863084	Omicron, 22B	BA.5	Human	Portugal	2022/05/10	Illumina	Borges et al.
EPI_ISL_12117745	Omicron, 22 C	BA.2.12.1	Human	USA	2022/04/07	Illumina	Howard et al.
MT582494	HKU-SZ-005b	Early isolate	Human	China	2020/01/11	Nanopore	Chan et al.
MN908947	Wuhan-Hu-1	Early isolate	Human	China	2019/12/-	Illumina	Wu et al.
MW555334	IND/Isolate 5844	NA	Human	India	2020/06/30	Illumina	Gulati et al.
MW555598	COVID-19/2020	NA	Human	India	2020/06/30	Illumina	Kumar et al.
MZ404503	COVI-VAC	NA	–	USA	NA	Sanger	Wang et al.

## Results and discussion

Analysis of more than 1600 isolates from India revealed two isolates with significant recombination signals. In the detection results of RDP, five methods, GENECONV, MaxChi, Chimaera, SiScan, 3Seq gave significant recombination positive signals (*p* < 0.01) (Table 2), and it was inferred that the recombination region was located within the S gene.

**Table 2:** Results of recombination detection

Methods	Average P-Value
GENECONV	4.364 × 10^− 3^
MaxChi	1.613 × 10^− 3^
Chimaera	2.067 × 10^− 4^
SiScan	7.863 × 10^− 20^
3Seq	3.256 × 10^− 4^

**Fig. 1:**
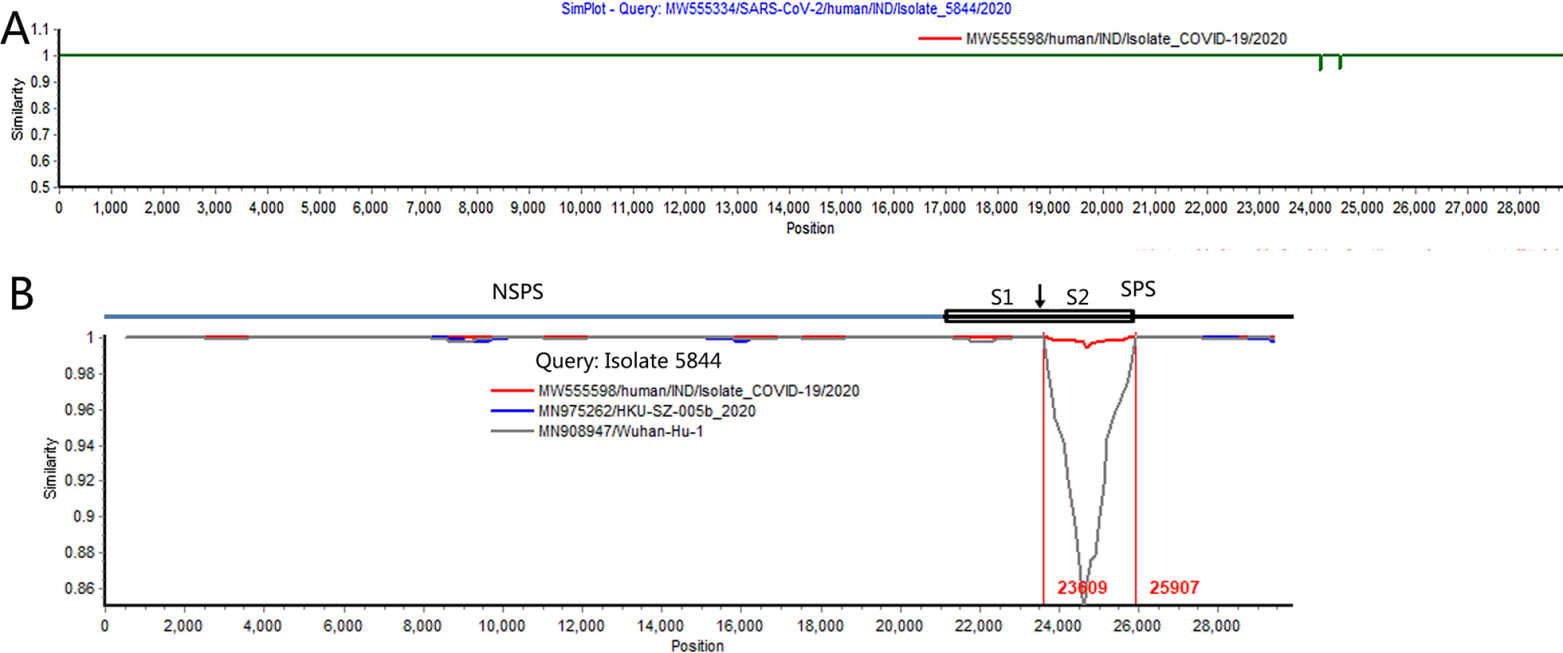
Comparison of genome sequence similarity between two Indian SARS-CoV-2s and the reference strains. **A** Genomic sequence comparison between two Indian isolates. The vertical axis is the sequence similarity between the viruses, and the horizontal axis is the corresponding position in the viral genome. The query sequence used for the comparison is the isolate 5844. **B** Comparison of genome sequence between the isolate 5844 and the reference strains Wuhan-Hu-1 and HKU-SZ-005b. The coding regions of the virus genome are shown with different colors, NSPS, non-structural protein, SPS, structural protein. The arrow points to the hydrolysis site of Furin enzyme

The two viruses with the recombination signal were isolated from two infected peoples on June 30, 2020. After removing the ambiguous bases, their genome sequences had 99.99% similarity, with only four bases different in the S gene (Fig. 1A). Comparing their genome sequences with the wild strains Wuhan-Hu-1 and HKU-SZ-005b isolated early in the virus outbreak, the two Indian isolates were almost identical (> 99.9%) to the two reference virus sequences except for the S gene. However, in the local region of the S gene, their similarity was less than 90% (Fig. 1B). This highly variable region is located in the S2 coding region (Fig. 1B). According to the evolution rate of SARS-CoV-2, the annual substitution rate of each site of the S gene is about 5.7 × 10^− 4^ [8]. Therefore, if the variation is caused by natural mutation, the S gene of these two Indian isolates might differ from other SARS-CoV-2 isolates by up to 3–4 bases at most. It means that the parent virus that can provide the S2 region for these two Indian isolates may not exist in nature. Therefore, the putative recombination regions on the genomes of the two Indian isolates should not originate from the recombination between SARS-CoV-2 circulating in nature, but are more likely to be the product of genetic engineering.

**Fig. 2:**
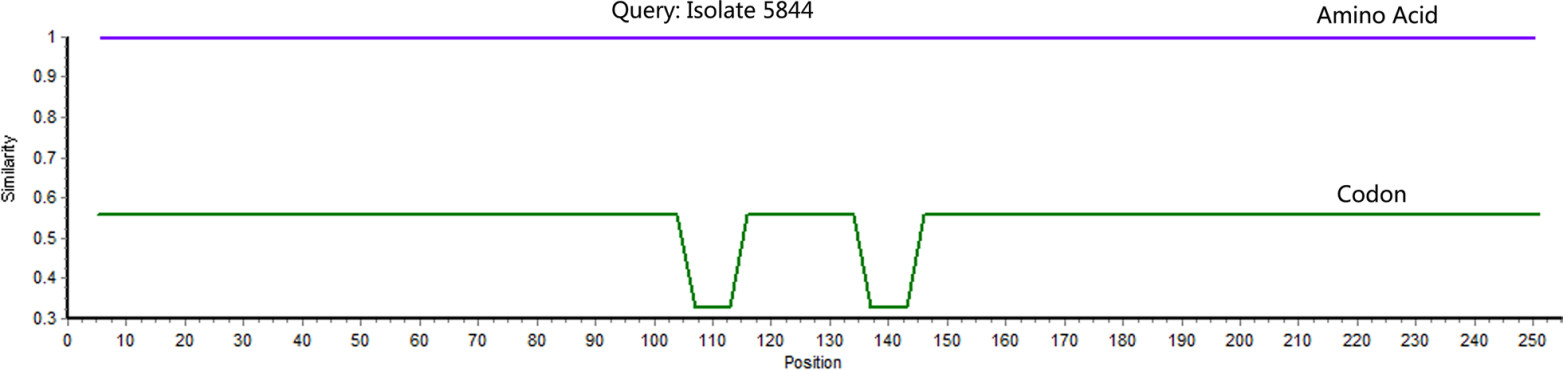
Comparison of amino acids (top) and codons (bottom) in the S2 region of Indian isolate 5844 and Wuhan-Hu-1. All ambiguous bases have been removed prior to sequence comparison, resulting in a final comparison of 78 codons (red) and their encoded amino acids (blue)

The substituted codon signature also indicated that the S2 region of these two Indian isolates was the product of artificial editing. We analyzed the substitution sites in S2 region of the isolate 5844 and found that after removal of ambiguous bases, there were nucleotide substitutions in codons of approximately 90 amino acids compared to the earliest SARS-CoV-2 isolate, Wuhan-Hu-1. Interestingly, although substitutions also appeared in the first position of very few codons, almost all substitutions occurred in the third position of these codons. Interestingly, all these substitutions took place between synonymous codons and did not change any amino acids of the S protein (Fig. 2). This regular substitution rule is significantly different from the natural mutation in S gene of SARS-CoV-2, and is more in line with LAV constructed by genetic recombination after artificial editing of the S2 region.

**Fig. 3:**
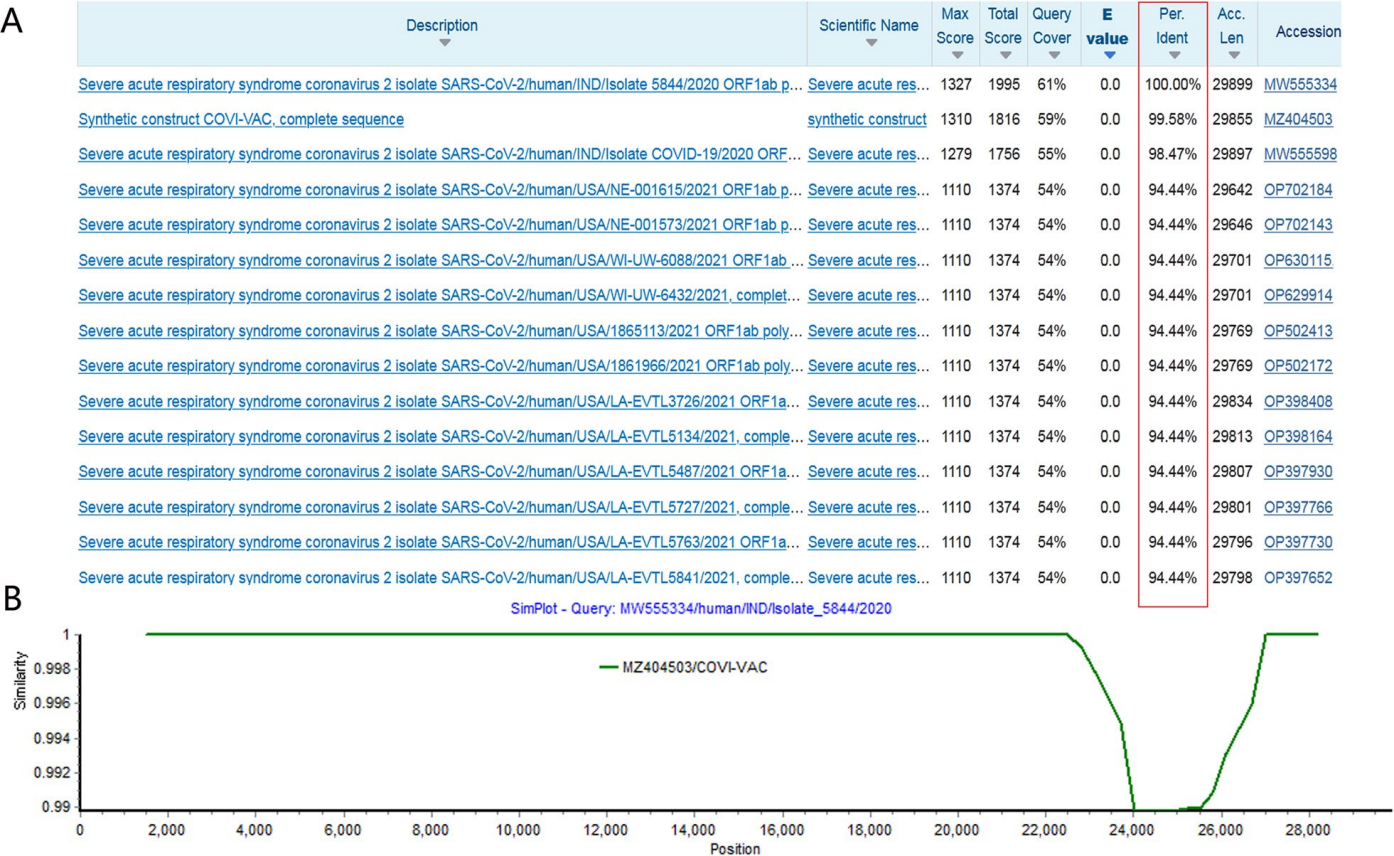
Sequence comparison between the Indian isolate 5844 and its homologous viruses. **A** Result of BLAST analysis performed in GenBank using the S2 region of the Indian isolate 5844 as the query (BLAST was performed on June 10, 2022). Sequence similarity between the isolate 5844 and other viruses was indicated by a red box. **B** Genome sequence comparison of the Indian isolate 5844 with the live attenuated vaccine strain COVI-VAC. The vertical axis is the sequence similarity between viruses, the horizontal axis is the position of the virus genome, and the query sequence used for comparison is the isolate 5844

Based on the above analysis, the two Indian SARS-CoV-2s are likely to be resulted from the spillover of LAV, rather than the product of natural recombination between circulating viruses. To test this hypothesis, using the S2 region of the isolate 5844 as the query sequence, we searched the SARS-CoV-2 database in GenBank to find the virus with the highest genomic similarity to them. It was found that the genomic sequence of the candidate LAV COVI-VAC, which was undergoing phase I clinical trials, had up to 99.6% similarity of the query virus. Until June 2022, with the exception of the vaccine strain COVI-VAC, we have not found any wild circulating strains that are more than 95% similar to the two India isolates in this region (Fig. 3A). This also suggested that the orthologous S gene of them was unlikely to have arisen through natural evolution of SARS-CoV-2. Further comparing the whole genomic sequence of the isolate 5844 with that of COVI-VAC, we found that their differences were in the S2 region, with a substitution of the total of 21 bases, while other regions had almost no changes. It was also noticed that, unlike Isolate_5844, the Furin enzyme cleavage site of COVI-VAC was missing (Fig. 3B). These results indicated that the two Indian strains might not be directly derived from COVI-VAC.

**Fig. 4:**
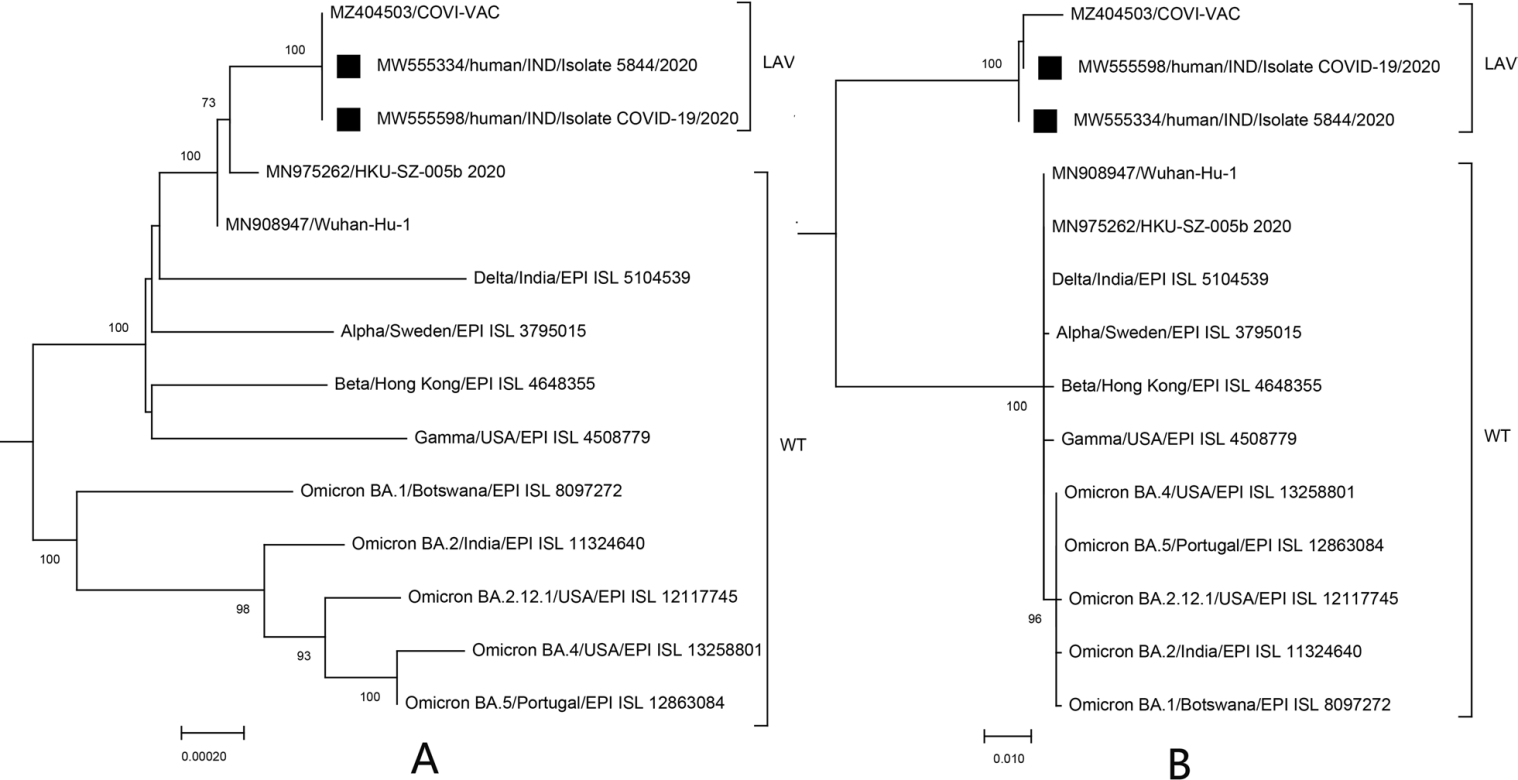
Phylogenetic histories of different regions of the genome of the two Indian isolates. **A** Phylogenetic tree inferred from positions 1-24105 of the SARS-CoV-2 genome; **B** Phylogenetic tree inferred from positions 24,106–25,300 (S2 region) of the SARS-CoV-2 genome. LAV, live attenuated vaccine; WT wild-type viruses

To demonstrate that these two Indian SARS-CoV-2 isolates may be live attenuated vaccine-derived strains, we reconstructed their phylogenetic histories. Phylogenetically, regardless of the S2 region of the genome or other regions, these two Indian isolates and the candidate LAV COVI-VAC formed a monophyletic group (Fig. 4), supporting that they should be spillover vaccine strains.

Although we cannot determine their real parents yet, the above results showed that the two SARS-CoV-2 isolates from India might be derived from the LAV candidate strains. Moreover, their S gene is most likely the product of genetic engineering after codon deoptimization. Fortunately, apart from these two Indian viruses, we have not found any more circulating viruses homologous to them in the SARS-CoV-2 databases so far, suggesting that these LAV-derived viruses have not spread widely among the population.

Before July 2021, LAVs were still at stages of laboratory research or phase I clinical trials [21]. The two Indian isolates were collected in June 2020, indicating that they may be the viruses spilled out during animal or clinical trials of LAVs, or resulted from outflow of laboratories. This finding suggested that there was a risk of spillover of LAVs into the environment, and therefore, may have some unpredictable consequences for SARS-CoV-2 control. The immediate impact will be to complicate SARS-CoV-2 surveillance. According to WHO recommendations, a positive real-time PCR result of viral nucleic acid test is the gold standard for determining whether someone is infected by SARS-CoV-2. However, if peoples are infected by the spilled LAV, they will also be test positive of nucleic acid, making it difficult to determine whether they are patients infected by wild SARS-CoV-2. Therefore, how to distinguish vaccine strains circulating in the environment from wild strains is one of the challenges faced by SARS-CoV-2 LAV development. In this sense, the construction of LAVs with gene deletion may be a good option to solve this problem.

Another issue posed by the spillover of LAVs is how to avoid reversion of the virulence of vaccine strains circulating in the environment. Theoretically, due to the use of multiple point mutations during the construction of LAVs, it is unlikely that SARS-CoV-2 will be resulted in virulence reversion through gene mutation. However, homologous recombination among viruses is the intrinsic genetic mechanism by which SARS-CoV-2 evolves rapidly [2, 17, 22, 27]. If homologous recombination occurs between wild viruses and vaccine strains circulating in the environment, there will be some unpredictable consequences. One lesson comes from the WHO Global Polio Eradication Program. Through the coverage of large-scale oral poliovirus vaccine, the global control of poliomyelitis has achieved good results, and almost completed the WHO goal of eradicating wild poliovirus [7, 29]. Unfortunately, over the course of several years in the early 2000s, Africa saw several outbreaks of polio associated with attenuated vaccination [10]. After in-depth research, it was found that these outbreaks were caused by the reversion of vaccine virulence because of the recombination of the spillover LAV with enteroviruses [9], which seriously interferes with the polio eradication plan. Therefore, how to avoid vaccine virus spillover and recombine with wild coronaviruses is also an issue that must be considered in the development of SARS-CoV-2 LAVs.

In conclusion, this study found that there might be some LAV-like strains among the SARS-CoV-2 strains circulating in the Indian population. In the phylogenetic trees inferred from different regions of the genome, they fall into the LAV lineage, and thus may be result from spillover of LAV. This finding suggests the risk of loss of live attenuated vaccines from vaccinated individuals into the environment, thereby increasing the complexity of SARS-CoV-2 control. In addition, recombination of attenuated vaccines with wild viruses may also have unforeseen consequences. Therefore, how to avoid recombination between vaccines circulating in the environment and wild strains is an important challenge during the research of LAVs.

## Data Availability

The data will be shared on a reasonable request to the corresponding author.

## References

[1] Alanagreh L, Alzoughool F, Atoum M (2020). The human coronavirus disease COVID-19: its origin, characteristics, and insights into potential drugs and its mechanisms. Pathogens.

[2] Amoutzias GD, Nikolaidis M, Tryfonopoulou E, Chlichlia K, Markoulatos P, Oliver SG (2022). The remarkable evolutionary plasticity of Coronaviruses by Mutation and recombination: insights for the COVID-19 pandemic and the future evolutionary paths of SARS-CoV-2. Viruses.

[3] Bao Y, Sun Y, Meng S, Shi J, Lu L (2020). 2019-nCoV epidemic: address mental health care to empower society. Lancet.

[4] Dumonteil E, Herrera C (2020). Polymorphism and selection pressure of SARS-CoV-2 vaccine and diagnostic antigens: implications for immune evasion and serologic diagnostic performance. Pathogens.

[5] Golawski M, Lewandowski P, Jablonska I, Delijewski M (2022). The reassessed potential of SARS-CoV-2 attenuation for COVID-19 Vaccine Development-A systematic review. Viruses.

[6] Grifoni A, Weiskopf D, Ramirez SI, Mateus J, Dan JM, Moderbacher CR, Rawlings SA, Sutherland A, Premkumar L, Jadi RS, Marrama D, de Silva AM, Frazier A, Carlin AF, Greenbaum JA, Peters B, Krammer F, Smith DM, Crotty S, Sette A (2020). Targets of T cell responses to SARS-CoV-2 coronavirus in humans with COVID-19 disease and unexposed individuals. Cell..

[7] Heymann DL (2004). Polio eradication: finishing the job and protecting the investment. Bull World Health Organ.

[8] Jo WK, Drosten C, Drexler JF (2021). The evolutionary dynamics of endemic human coronaviruses. Virus Evol.

[9] Kew O, Morris-Glasgow V, Landaverde M, Burns C, Shaw J, Garib Z, Andre J, Blackman E, Freeman CJ, Jorba J, Sutter R, Tambini G, Venczel L, Pedreira C, Laender F, Shimizu H, Yoneyama T, Miyamura T, van Der Avoort H, Oberste MS, Kilpatrick D, Cochi S, Pallansch M, de Quadros C (2002). Outbreak of poliomyelitis in Hispaniola associated with circulating type 1 vaccine-derived poliovirus. Science.

[10] Kew OM, Wright PF, Agol VI, Delpeyroux F, Shimizu H, Nathanson N, Pallansch MA (2004). Circulating vaccine-derived polioviruses: current state of knowledge. Bull World Health Organ.

[11] Kumar S, Stecher G, Li M, Knyaz C, Tamura K (2018). MEGA X: molecular evolutionary genetics analysis across computing platforms. Mol Biol Evol.

[12] Lai MM (1990). Coronavirus: organization, replication and expression of genome. Annu Rev Microbiol.

[13] Lai MM, Cavanagh D (1997). The molecular biology of coronaviruses. Adv Virus Res.

[14] Li Q, Guan X, Wu P, Wang X, Zhou L, Tong Y, Ren R, Leung KSM, Lau EHY, Wong JY, Xing X, Xiang N, Wu Y, Li C, Chen Q, Li D, Liu T, Zhao J, Liu M, Tu W, Chen C, Jin L, Yang R, Wang Q, Zhou S, Wang R, Liu H, Luo Y, Liu Y, Shao G, Li H, Tao Z, Yang Y, Deng Z, Liu B, Ma Z, Zhang Y, Shi G, Lam TTY, Wu JT, Gao GF, Cowling BJ, Yang B, Leung GM, Feng Z (2020). Early transmission dynamics in Wuhan, China, of novel coronavirus-infected pneumonia. New Engl J Med.

[15] Li Q, Wang J, Tang Y, Lu H (2021). Next-generation COVID-19 vaccines: opportunities for vaccine development and challenges in tackling COVID-19. Drug Discov Ther.

[16] Liu Y, Zhang X, Liu J, Xia H, Zou J, Muruato AE, Periasamy S, Plante JA, Bopp NE, Kurhade C, Bukreyev A, Ren P, Wang T, Vineet DM, Plante KS, Xie X, Weaver SC, Shi PY. A live-attenuated SARS-CoV-2 vaccine candidate with accessory protein deletions. bioRxiv. (2022). 10.1038/s41467-022-31930-z PMC9326133 35896528

[17] Lohrasbi-Nejad A (2022). Detection of homologous recombination events in SARS-CoV-2. Biotechnol Lett.

[18] Lole KS, Bollinger RC, Paranjape RS, Gadkari D, Kulkarni SS, Novak NG, Ingersoll R, Sheppard HW, Ray SC (1999). Full-length human immunodeficiency virus type 1 genomes from subtype C-infected seroconverters in India, with evidence of intersubtype recombination. J Virol.

[19] Maitra A, Sarkar MC, Raheja H, Biswas NK, Chakraborti S, Singh AK, Ghosh S, Sarkar S, Patra S, Mondal RK, Ghosh T, Chatterjee A, Banu H, Majumdar A, Chinnaswamy S, Srinivasan N, Dutta S, Das S (2020). Mutations in SARS-CoV-2 viral RNA identified in Eastern India: possible implications for the ongoing outbreak in India and impact on viral structure and host susceptibility. J Biosci.

[20] Martin DP, Williamson C, Posada D (2005). RDP2: recombination detection and analysis from sequence alignments. Bioinformatics.

[21] Motamedi H, Ari MM, Dashtbin S, Fathollahi M, Hossainpour H, Alvandi A, Moradi J, Abiri R (2021). An update review of globally reported SARS-CoV-2 vaccines in preclinical and clinical stages. Int Immunopharmacol.

[22] Nikolaidis M, Papakyriakou A, Chlichlia K, Markoulatos P, Oliver SG, Amoutzias GD (2022). Comparative analysis of SARS-CoV-2 variants of concern, including omicron, highlights their common and distinctive amino acid substitution patterns, especially at the spike ORF. Viruses.

[23] Nugent MA (2022). The future of the COVID-19 pandemic: How good (or bad) can the SARS-CoV2 spike protein get?. Cells.

[24] Seo SH, Jang Y (2020). Cold-adapted live attenuated SARS-Cov-2 vaccine completely protects human ACE2 transgenic mice from SARS-Cov-2 infection. Vaccines (Basel).

[25] Souza PFN, Mesquita FP, Amaral JL, Landim PGC, Lima KRP, Costa MB, Farias IR, Belem MO, Pinto YO, Moreira HHT, Magalhaes ICL, Castelo-Branco D, Montenegro RC, de Andrade CR (2022). The spike glycoprotein of SARS-CoV-2: a review of how mutations of spike glycoproteins have driven the emergence of variants with high transmissibility and immune escape. Int J Biol Macromol.

[26] Trimpert J, Dietert K, Firsching TC, Ebert N, Thao TN, Vladimirova T, Kaufer D, Labroussaa S, Abdelgawad F, Conradie A, Hofler A, Adler T, Bertzbach JM, Jores LD, Gruber J, Thiel AD, Osterrieder V, Kunec N (2021). Development of safe and highly protective live-attenuated SARS-CoV-2 vaccine candidates by genome recoding. Cell Rep.

[27] Wang W, Li CP, He M, Li SW, Cao L, Ding NZ, He CQ (2021). The dominant strain of SARS-CoV-2 is a mosaicism. Virus Res.

[28] Wang Y, Yang C, Song Y, Coleman JR, Stawowczyk M, Tafrova J, Tasker S, Boltz D, Baker R, Garcia L, Seale O, Kushnir A, Wimmer E, Mueller S (2021). Scalable live-attenuated SARS-CoV-2 vaccine candidate demonstrates preclinical safety and efficacy. Proc Natl Acad Sci U S A.

[29] World-Health-Organization (2003). Progress towards global eradication of poliomyelitis, 2002. Relev Epidemiol Hebd.

[30] Zhu N, Zhang D, Wang W, Li X, Yang B, Song J, Zhao X, Huang B, Shi W, Lu R, Niu P, Zhan F, Ma X, Wang D, Xu W, Wu G, Gao GF, Tan W, China Novel Coronavirus, Research I, T. A novel coronavirus from patients with pneumonia in China, 2019. New Engl J Med. 2020;382:727–733. 10.1056/NEJMoa2001017 PMC7092803 31978945

